# Exploring the Influence of Object Similarity and Desirability on Children’s Ownership Identification and Preferences in Autism and Typical Development

**DOI:** 10.1007/s10803-022-05489-z

**Published:** 2022-03-23

**Authors:** Calum Hartley, Laura-Ashleigh Bird

**Affiliations:** 1 Department of Psychology, Lancaster University, Lancaster, LA1 4YF UK; 2 Institute of Cognitive Neuroscience, University College London, London, UK

**Keywords:** Autism Spectrum Disorder, Ownership, Owner identification, Object preferences

## Abstract

This study investigated how ownership identification accuracy and object preferences in children with autism spectrum disorder (ASD) are influenced by visual distinctiveness and relative desirability. Unlike typically developing (TD) children matched on receptive language (*M* age equivalents: 58.8–59.9 months), children with ASD had difficulty identifying another person’s property when object discriminability was low and identifying their own relatively undesirable objects. Children with ASD identified novel objects designated to them with no greater accuracy than objects designated to others, and associating objects with the self did not bias their preferences. We propose that, due to differences in development of the psychological self, ownership does not increase the attentional or preferential salience of objects for children with ASD.

**Supplementary Information:**

The online version contains supplementary material available at 10.1007/s10803-022-05489-z.

## Introduction

Identifying ‘who owns what’ is a crucial foundation of children’s social development. However, this task is complicated by the fact that ownership is a cognitive construction rather than a visible object quality. Accurate detection of ownership is contingent on tracking invisible associations between people and their property. By the time they reach preschool, typically developing (TD) children are highly sensitive to ownership history and recognise the distinction between liking and owning an object (Nancekivell & Friedman, 2014; Noles & Gelman, 2014). Ownership also influences TD children’s preferences for objects – they often consider their self-owned objects to be more valuable and desirable than similar non-owned objects (Gelman & Echelbarger, 2019; Gelman et al., 2012; Hood & Bloom, 2008). Conversely, recent research has shown that children with autism spectrum disorder (ASD) may be less accurate at identifying ownership (Hartley et al., 2021) and that their object preferences are not influenced by ownership history (Hartley & Fisher, 2018; Hartley et al., 2020). Here, we investigate how the perceptual similarity and relative desirability of objects impacts ownership identification accuracy and object preferences in children with ASD.

TD children can accurately match people to property even when object discriminability is very low and ownership is at odds with desirability. In Gelman et al. (2012), 2-year-olds, 3-year-olds, and adults were presented with sets of three objects; one object was assigned to the participant and another was assigned to an experimenter. Participants were then asked to match the objects to their corresponding owners. In ‘varied’ trials, the three objects were perceptually distinct and similarly desirable (e.g. three different toy vehicles), meaning that children could succeed by associating unique objects with different owners. In ‘identical’ trials, the three objects were exactly the same (e.g. three indistinguishable toy taxis), meaning that accurate owner identification required children to track invisible associations between people and specific exemplars of a single object type. In ‘participant-plain’ trials, the three objects were perceptually distinct, but the child’s object was relatively undesirable in comparison to the other objects (e.g. a boring wooden cube vs. a toy train and toy motorcycle). These trials tested participants’ understanding that they can own an object that they dislike, and that desiring another’s object does not confer ownership to the self. Adults and 3-year-olds identified their objects and the experimenter’s objects with above-chance accuracy in each trial type. The 2-year-olds identified their own objects and the experimenter’s objects with above-chance accuracy in varied trials, and the experimenter’s objects in participant-plain trials, but struggled to identify themselves as the owner of relatively boring objects and could not accurately match objects to either party in identical trials. Thus, although TD children can accurately match objects to owners based purely on historical associations from 3 years, object similarity and differences in object desirability may hinder owner-object tracking for children with less-developed understanding of ownership.

In addition to assessing owner identification accuracy, Gelman et al. (2012) also asked participants to indicate which objects they and the experimenter preferred. It is well-documented that neurotypical children consider their property to be more valuable, desirable, and memorable than similar non-owned property (Harbaugh et al., 2001; Hartley & Fisher, 2018; Hood & Bloom, 2008). Establishing ownership forges a mentalistic connection between a person and an object, and because people tend to view themselves favourably, this can trigger the transfer of positive self-perceptions to self-owned property (Hood et al., 2016). Accordingly, Gelman et al (2012) found that their participants aged 2 and 3 years preferred their designated objects above-chance in varied and identical trials, but not participant-plain trials (which is unsurprising given the relative undesirability of their objects). However, 2- and 3-year-olds only inferred that the experimenter preferred their assigned item at above-chance rates in participant-plain trials. By contrast, adults only preferred their designated objects above-chance in identical trials, but inferred that the experimenter preferred their designated objects above-chance across all trial types. Together, these findings demonstrate that merely establishing ownership is sufficient to induce preferential biases for randomly allocated objects in neurotypical pre-schoolers when ownership is not at odds with desirability. However, preschool children did not assume that other people preferred their owned items when object desirability was controlled, potentially reflecting limitations in mentalistic reasoning (Wellman et al., 2001).

ASD is characterised by pervasive differences in social skills (APA, 2013), and children with this condition often spend less time interacting with others (McConnell, 2002). As ownership is a cultural convention acquired through social interactions (Kanngiesser et al., 2015), it may be that children with ASD differ in their development of ownership understanding, which may in turn contribute to their difficulties in social settings. Research has only recently begun to investigate the impact of ASD on children’s understanding of ownership, with just two studies to date examining owner identification accuracy. In Hartley and Fisher (2018), TD children and children with ASD matched on receptive vocabulary (~ 4.8 years) were randomly assigned one of three visually distinct toys to keep, before being offered the chance to trade for a preferred alternative. The remaining two objects were designated to the experimenter and a puppet. When asked to identify the owner of each object, both populations achieved comparably high accuracy for self- and other-owned objects. In Hartley et al. (2021), similar samples of vocabulary-matched children with ASD and TD controls were presented with sets of three objects of the same type (e.g. three differently-coloured drinking bottles) – one object belonged to the child and two objects belonged to experimenters – and were asked to identify the owner of each object. While both groups identified their own objects with similar accuracy, children with ASD were significantly less accurate at identifying objects belonging to other owners than TD children.

One explanation for the reduced owner identification accuracy of children with ASD in Hartley et al. (2021) relates to the objects’ perceptual distinctiveness. Unlike in Hartley and Fisher (2018), the three objects belonged to the same category and shared similarities in global shape. Thus, it may be that owner identification accuracy in ASD is increasingly dependent on perceptual discriminability. Whereas TD 3-year-olds are highly sensitive to what they and others own (Pesowski et al., 2019), ASD may reduce children’s attention to ownership history, particularly when tracking relationships between other owners and their property. In addition to testing this hypothesis, the present research also explores the influence of object desirability on owner identification accuracy in children with ASD. If understanding of ownership is delayed in ASD due to early differences in social-cognitive development, it is possible that object desirability may confound their tracking of owner-object associations as observed in TD 2-year-olds (Gelman et al., 2012). Indeed, difficulty separating the concepts of ownership and desire would have important implications for children’s navigation of social situations with peers and potentially cause inadvertent breaches of others’ ownership rights.

Hartley and Fisher (2018) also collected data pertaining to the influence of ownership on object preferences for children with ASD. While TD children demonstrated a “mere ownership effect” by showing a reliable preference for their randomly assigned toy and rarely trading, children with ASD often traded for an alternative toy that they liked more. Furthermore, unlike TD children, children with ASD did not assign significantly higher values to self-selected or randomly assigned toys in comparison to different non-owned toys or identical copies. More recently, Hartley et al. (2020) found that children with ASD did not over-value authentic items with special ownership histories (e.g. SpongeBob’s spatula) in comparison to similar inauthentic objects belonging to non-famous owners (e.g. my brother’s whisk), whereas TD children assigned significantly higher values based on authentic ownership history. However, TD children and children with ASD did not differ in their sensitivity to object qualities unrelated to ownership (e.g. newness and rarity) as determinants of value. Taken together, these findings suggest that ownership history in relation to the self or other special owners does not influence object preferences for children with ASD (for further evidence concerning the absence of ‘self-reference’ effects in ASD, see Gillespie-Smith et al. (2018), Nijhof and Bird (2019), and Wuyun et al. (2020)). Rather, children with ASD may be principally concerned with an object’s qualities (i.e. what an object is) rather than whom it belongs to.

As prior studies have focused on examining the influence of ownership on children’s object preferences, it is currently unknown how children with ASD perceive ownership to influence *others’* object preferences. TD children understand that other people may prefer non-owned objects if they are more desirable than self-owned objects by 5 years (Pesowski & Friedman, 2018) and, as their understanding of ownership matures, are likely to infer that other owners prefer their property when ownership and desirability are not at odds (Gelman et al., 2012). Such judgements require the ability to take the perspective of another agent and generalise knowledge of how ownership influences one’s own object preferences. Given that differences in mentalistic reasoning are a common characteristic of ASD (Altschuler et al., 2018; Berenguer et al., 2018), and recent evidence suggests that children with ASD are less likely to defend the ownership rights of others (Hartley et al., 2021), the assumption that other owners hold preferences for their property may be developmentally delayed.

The objective of this study was to investigate how ownership identification and object preferences in children with ASD are influenced by visual distinctiveness and differences in desirability. Vocabulary-matched children with ASD and TD controls completed a slightly modified version of Gelman et al.’s (2012) task, in which they were presented with sets of three objects and asked to identify their owners and indicate which objects they and an experimenter preferred. Object sets corresponded to either varied, identical, or participant-plain trials as described above. Based on previous evidence, we predicted that children with ASD would be less accurate than TD children at identifying owners when objects are visually similar and, potentially, when objects differ in desirability. We also predicted that ASD would suppress preferences for objects associated with the self across trial types, and that children with ASD would be less likely to infer that the experimenter preferred items designated to them. The results of this study will advance theoretical knowledge by providing new insight into how fundamental aspects of ownership cognition are influenced by ASD.

## Methods

### Participants

Participants were 20 children with ASD (*M* age = 7.73 years, *SD* = 4.47, range = 5.75–11.17 years, 16 males) and 20 TD children (*M* age = 4.70 years, *SD* = 1.06, range = 3.25–7.67 years, 12 males) recruited from specialist schools, mainstream schools, and preschools. Samples were closely matched on receptive vocabulary as measured by the British Picture Vocabulary Scale (BPVS; Dunn et al., 1997; ASD *M* age equivalent = 4.90 years, *SD* = 1.20, range 3.00–6.58 years; TD *M* age equivalent = 4.99 years, *SD* = 0.88, range 3.42–6.58 years), *t*(38) = 0.26, *p* = 0.79. All children with ASD were diagnosed by a qualified educational or clinical psychologist using standardised instruments (e.g. Autism Diagnostic Observation Scale and Autism Diagnostic Interview-Revised; Lord et al., 1994, 2002) and expert judgement. Diagnoses were confirmed via the Childhood Autism Rating Scale 2 (CARS; Schopler et al., 2010), which was completed by each participant’s class teacher (ASD *M* score: 34.90; TD *M* score: 15.68). Children with ASD were significantly older than TD children *t*(38) = 7.47, *p* < 0.001, *d* = 2.36, and had significantly higher CARS scores, *t*(38) = 14.11, *p* < 0.001, *d* = 4.46. All procedures performed in this research involving human participants were in accordance with the ethical standards of the institutional and national research committees. Informed consent was obtained from parents/caregivers prior to children’s participation.

### Materials

**Fig. 1:**
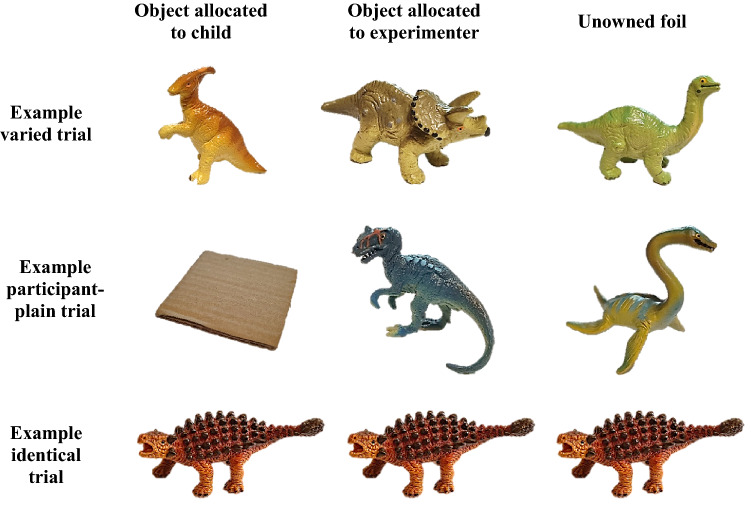
Example stimuli and trial types corresponding to the dinosaur thematic category

Materials included four groups of 9 objects. Each group contained 8 objects belonging to a thematic category (model dinosaurs, animals, vehicles, and furniture), plus one “plain/boring” object (e.g. a cardboard square). Three of these thematic categories – animals, furniture, and vehicles – were selected to match those used in Gelman et al. (2012), thus providing a high degree of comparability. The fourth thematic category employed by Gelman and colleagues, model items of food, was replaced with dinosaurs because many children with ASD have dietary restrictions and aversions that could have potentially biased their responding in the experimental task (Mayes & Zickgraf, 2019). The thematic objects were matched on size, complexity, and attractiveness within their group. The plain objects were selected on the basis that they were comparatively less interesting and colourful than the thematic objects. Within each group, the objects were organised into three sets corresponding to different trial types: varied, participant-plain, and identical. The ‘varied’ set contained three different thematic objects (e.g. three different model animals). The ‘participant-plain’ group contained two different thematic objects, plus one of the plain objects (e.g. two different model animals and a small wooden circle). The ‘identical’ group contained three perceptually identical thematic objects (e.g. three identical model animals). Example stimuli and trial types are illustrated in Fig. 1.

### Procedure

Children completed the BPVS and the experimental task on different days, approximately one week apart. For the experimental task, children sat at a table opposite the experimenter and were invited to play a game with some toys. On each trial, three objects were presented individually. The experimenter held up each object in turn and drew attention to it with, or without, referencing ownership status. One object was placed in front of the child and designated as belonging to them (e.g. “This is yours, this is for [child’s name]”). Another object was placed in front of the experimenter and designated to them (e.g. “This is mine, this is for Laura”). The remaining object was placed equidistant between the child and the experimenter, and was not assigned an owner (e.g. “See this, look at this”). These three object types were presented in four counterbalanced orders, ensuring that the foil never separated the self-target and other-target (1. Self, Other, Foil; 2. Other, Self, Foil; 3. Foil, Self, Other; 4. Foil, Other, Self). Presenting the foil between the two targets may have reduced the distinction between them. The three objects were then gathered together and positioned in a row in the middle of the table, with each item an equal distance from the child and experimenter. Location of each object type in the array (middle, left, right) was counterbalanced.

Children were asked four test questions. The first pair of questions concerned object ownership; children were asked to identify (a) the object that was assigned to them (e.g. “Which one is yours? Which belongs to [child’s name]?”), and (b) the object assigned to the experimenter (e.g. “Which one is mine? Which belongs to Laura?”). The order of these ownership questions was counterbalanced across trials. The second pair of questions concerned object preferences; children were asked to identify (a) the object they preferred (e.g. “Which one do you like best? Which is [child’s] favourite?”), and (b) the object the experimenter preferred (e.g. “Which one do I like best? Which is Laura’s favourite?”). The order of these preference questions was counterbalanced across trials. Following the child’s response to the fourth test question, the three objects were removed and the next trial was initiated.

Children completed 12 trials in total; four ‘varied’ trials involving three different thematic objects, four ‘participant-plain’ trials involving two different thematic objects and one relatively boring object, and four ‘identical’ trials involving three identical thematic objects. Children received one trial of each type with stimuli corresponding to each of the four thematic categories (dinosaurs, animals, vehicles, furniture), and no objects were reused across trials. Object assignment to the child was randomised in the variable and identical trials, but not participant-plain trials (they were always assigned the boring, less desirable, non-thematic object). Object assignment to the experimenter was randomised across trial types. The order of trials was pseudo-randomised with the constraint that no more than two trials of the same type, or belonging to the same thematic set, were experienced consecutively. In total, the game lasted ~ 10 min.

## Results

Children’s responses to each of the four test questions were coded 0 or 1 (yielding a total score of 4 per question). For the owner identification questions, children scored 1 if they correctly selected the objects that were assigned to them or the experimenter. When asked which object they preferred, children scored 1 if they selected the object that was assigned to them (indicating a mere ownership effect). When asked to identify the experimenter’s preferred object, children scored 1 if they selected the object that was assigned to the experimenter (thus inferring that they would display a mere ownership effect).

### Owner Identification Accuracy

**Fig. 2:**
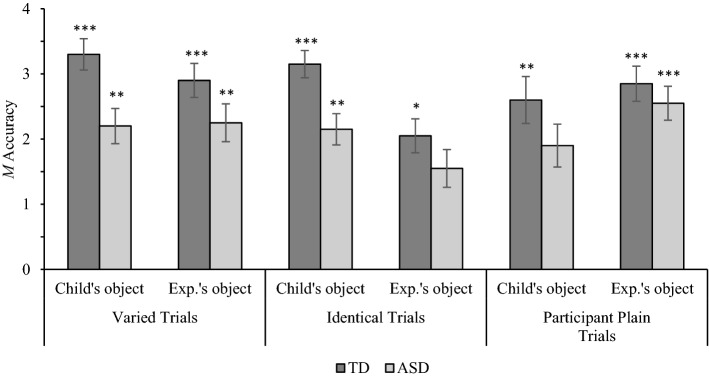
Mean owner identification accuracy for typically developing (TD) children and children with autism spectrum disorder (ASD) in varied, identical, and participant plain trials. Error bars show ± 1 SE. Stars above columns indicate where accuracy significantly exceeded chance (1.32; * *p* < .05, ** *p* < .01, *** p < .001)

As there were three objects in each set, there was a 1 in 3 chance that children would select the correct object at random in response to an owner identification question. Summed across four trials per trial type, participants would be expected to correctly identify the participant’s object and experimenter’s object on 1.32 trials each by chance. In comparison to this chance value, one-sample *t*-tests showed that TD children identified their objects and the experimenter’s objects with above-chance accuracy across trial types (*p* = 0.01 to < 0.001; see Fig. 2). By contrast, children with ASD identified their objects with above-chance accuracy in varied (*p* = 0.004) and identical trials (*p* = 0.003), but not participant-plain trials (*p* = 0.096). Children with ASD identified the experimenter’s objects with above-chance accuracy in varied (*p* = 0.005) and participant-plain trials (*p* < 0.001), but not identical trials (*p* = 0.43).

The owner identification responses were entered into a 2(Population: ASD, TD) × 3(Trial Type: varied, identical, participant-plain) × 2(Owner: child, experimenter) mixed ANOVA (descriptive statistics are displayed in Fig. 2). A significant main effect of Trial Type, *F*(2, 76) = 3.86, MSE = 1.00, *p* = 0.025, η_p_^2^ = 0.092, was qualified by a significant Trial Type x Owner interaction, *F*(2, 76) = 15.04, *p* < 0.001, η_p_^2^ = 0.28. Pairwise comparisons showed that, across populations, children identified their objects significantly more accurately than the experimenter’s objects in identical trials (*p* < 0.001), but identified the experimenter’s objects significantly more accurately than their objects in participant-plain trials (*p* = 0.02). There was no effect of Owner on participants’ owner identification accuracy in Varied trials (*p* = 0.23). A repeated measures ANOVA showed a significant effect of Trial Type on participants’ owner identification accuracy for their objects, *F*(2, 78) = 3.32, MSE = 0.84, *p* = 0.041, η_p_^2^ = 0.079. Pairwise comparisons showed that children identified their objects significantly more accurately in varied trials than participant-plain trials (*p* = 0.012). No other between-trial differences were detected (varied vs. identical: *p* = 1.00; participant-plain vs. identical: *p* = 0.34). Another repeated measures ANOVA revealed a significant effect of Trial Type on participants’ owner identification accuracy for the experimenter’s objects, *F*(2, 78) = 13.65, MSE = 0.70, *p* < 0.001, η_p_^2^ = 0.26. Participants identified the experimenter’s object significantly less accurately in identical trials than in varied trials (*p* = 0.001) and participant-plain trials (*p* < 0.001), which did not significantly differ (*p* = 1.00).

A significant main effect of Population, *F*(1, 38) = 5.78, MSE = 5.21, *p* = 0.021, η_p_^2^ = 0.13, was qualified by a borderline Population x Owner interaction, *F*(1, 38) = 3.90, MSE = 0.78, *p* = 0.056, η_p_^2^ = 0.093. Given our a priori hypotheses, we proceeded to deconstruct the relationship between Population and Owner, though note that the comparisons should be treated with caution as the interaction was marginally significant. Pairwise comparisons showed that TD children identified their objects with significantly greater accuracy than children with ASD (*p* = 0.005), but their owner identification accuracy for the experimenter’s objects did not significantly differ (*p* = 0.13). While children with ASD identified their objects and the experimenter’s objects with similar accuracy (*p* = 0.86), TD children identified their objects significantly more accurately than the experimenter’s (*p* = 0.004).

Pearson’s correlations were conducted to explore relationships between owner identification accuracy and measures of individual differences. Given our detection of between-population differences, relationships between variables were measured for each population separately. For the ASD group, ownership identification accuracy did not correlate with receptive vocabulary or chronological age. For the TD group, a significant relationship was detected between chronological age and children’s identification of their own object in identical trials (*R* = 0.45, *p* = 0.047). No other significant relationships were observed for TD children.

### Object Preferences

**Fig. 3:**
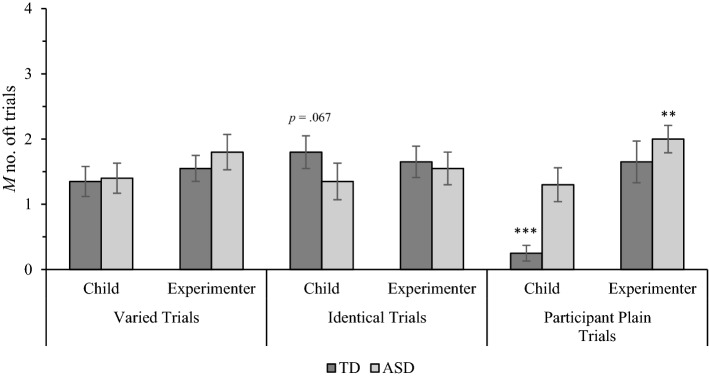
Mean number of trials that typically developing (TD) children and children with autism spectrum disorder (ASD) indicated that owners preferred their assigned objects in varied, identical, and participant plain trials. Error bars show ± 1 SE. Stars above columns indicate where responding significantly differed from chance (1.32; ** *p* < .01, *** *p* < .001)

As for owner identification accuracy, there was a 1 in 3 chance that children would select each object at random. Summed across four trials per trial type, participants would be expected to select their assigned item and the experimenter’s assigned items in response to corresponding preference questions on 1.32 trials each by chance. In comparison to this chance value, one-sample *t*-tests showed that TD children displayed a borderline-significant tendency to prefer their assigned objects in identical trials (*p* = 0.067; see Fig. 3). TD children’s preferences for their assigned objects did not differ from chance in varied trials (*p* = 0.90) and were significantly below-chance in participant-plain trials (*p* < 0.001). Interestingly, TD children did not infer that the experimenter preferred their assigned items at above-chance rates in any trial type (*p* = 0.19 to 0.31). The preferences of children with ASD for their assigned objects did not significantly differ from chance in any trial type (*p* = 0.74 to 0.94). Children with ASD inferred that the experimenter preferred their assigned items at above-chance rates in participant-plain trials (*p* = 0.004), but not varied (*p* = 0.09) or identical trials (*p* = 0.36).

The object preference responses were also analysed via a 2(Population: ASD, TD) × 3(Trial Type: varied, identical, participant-plain) × 2(Owner: child, experimenter) mixed ANOVA (descriptive statistics are displayed in Fig. 3). A significant main effect of Owner, *F*(1, 38) = 9.54, MSE = 1.32, *p* = 0.004, η_p_^2^ = 0.20, was qualified by a significant Trial Type x Owner interaction, *F*(1, 76) = 6.67, MSE = 0.85, *p* = 0.002, η_p_^2^ = 0.15. A repeated measures ANOVA showed that children were significantly less likely to prefer their assigned object in participant-plain trials than varied trials (*p* = 0.021) and identical trials (*p* = 0.013), which did not significantly differ (*p* = 0.97). Another repeated measures ANOVA showed that children’s likelihood of inferring that the experimenter preferred their assigned object did not differ across trial types (*p* = 0.53). Pairwise comparisons showed that, in participant-plain trials, children were significantly less likely to prefer their assigned object than infer that the experimenter preferred their assigned object (*p* < 0.001). Likelihood of children preferring their assigned object and inferring that the experimenter preferred their assigned object did not significantly differ in varied trials (*p* = 0.18) or identical trials (*p* = 0.92).

The three-way ANOVA examining children’s object preference responses also yielded a significant Population x Trial Type interaction, *F*(2, 76) = 5.44, MSE = 0.88, *p* = 0.006, η_p_^2^ = 0.13. Pairwise comparisons showed that children with ASD were significantly more likely to indicate that owners preferred their assigned objects in the participant-plain trials than TD children (*p* = 0.011). The populations did not significantly differ in varied (*p* = 0.55) or identical trials (*p* = 0.32). For TD children, a repeated measures ANOVA revealed a significant effect of Trial Type, *F*(2, 38) = 7.19, MSE = 0.43, *p* = 0.002, η_p_^2^ = 0.27. Pairwise comparisons showed that TD children were less likely to indicate that owners preferred their assigned objects in participant-plain trials than identical trials (a significant difference, *p* = 0.01) and varied trials (a borderline difference, *p* = 0.069). The difference between varied and identical trials did not approach significance (*p* = 0.47). Another repeated measures ANOVA showed that, for children with ASD, likelihood of indicating that owners preferred their assigned objects did not significantly differ across trial types (*p* = 0.62).

As for owner identification accuracy, relationships between children’s object preferences and individual difference measures were examined for each population separately using Pearson’s correlations. For the ASD group, a marginally significant relationship was detected between receptive vocabulary and the likelihood of children preferring their assigned object in varied trials (*R* = 0.44, *p* = 0.05). No other significant relationships were observed for children with ASD. For the TD group, children’s object preferences did not correlate with receptive language or chronological age.

## Discussion

This study investigated how object discriminability and differences in desirability influence ownership identification and preferences in children with ASD and TD children. While TD children identified their objects and the experimenter’s objects with above-chance accuracy across trial types, children with ASD had difficulty identifying their objects in participant-plain trials and the experimenter’s objects in identical trials. Across trial types, children with ASD were significantly less accurate than TD children at identifying their objects, but not the experimenter’s objects. In keeping with prior research, children with ASD preferred their assigned objects at chance rates in each trial type. By comparison, TD children showed a tendency to prefer their assigned objects in identical trials, and very rarely preferred their relatively undesirable objects in participant-plain trials. Neither group inferred that the experimenter would prefer their assigned items, with the exception of children with ASD in participant-plain trials. Collectively, these results suggest that children with ASD are less accurate at tracking owner-object relationships, and that associations with the self do not increase the attentional or preferential salience of objects for this population.

Much like the 3-year-old and adult participants in Gelman et al. (2012), our TD control group successfully matched objects to themselves and other owners with above-chance accuracy. However, the response profile of our children with ASD resembled that of TD 2-year-olds; they identified owners with above-chance accuracy in varied trials, but struggled to identify themselves as owners of relatively boring objects and keep track of the experimenter’s objects when they were not visually distinctive. On average, our children with ASD were aged 7.7 years with a mean receptive vocabulary age of 4.9 years, clearly exceeding the age at which TD pre-schoolers attain above-chance accuracy across owners and trial types (Gelman et al., 2012). This suggests that ASD may be characterised by an atypical developmental trajectory whereby high sensitivity to ownership history as a heuristic for accurately matching people to property emerges unusually late.

Perhaps owing to their differences in social-cognition and social motivation (APA, 2013; Chevallier et al., 2012), children with ASD appear to have greater difficulty detecting invisible ownership associations between people and property, particularly when they are unable to associate unique objects with different owners. Indeed, for children in both populations, the visual similarity of objects in identical trials hindered identification of the experimenter’s objects, whereas perceptual distinctiveness in varied and participant-plain trials had a facilitative effect. The finding that participants identified the experimenter’s objects significantly more accurately than their objects in participant-plain trials appeared to be most prominent in the ASD sample (TD children identified self- and other-owned objects in participant-plain trials with 65% and 71% accuracy respectively, whereas the corresponding scores for children with ASD were 47.5% and 64%). This suggests that children with ASD may have difficulty acknowledging their ownership of relatively undesirable objects, possibly indicating that differences in desirability may confound their tracking of owner-object relationships.

Our owner identification analyses also showed that children with ASD identified their objects and the experimenter’s objects with similar accuracy across trial types, whereas TD children identified their objects significantly more accurately than the experimenter’s objects. These findings show that, for TD children, merely associating novel objects with the self is sufficient to increase their attentional salience, resulting in greater tracking accuracy. This effect aligns with previous studies demonstrating that objects associated with the self are memorised and recalled more accurately than objects associated with others in both TD children (Cunningham et al., 2013) and adults (Cunningham et al., 2008). Conversely, for children with ASD, associating objects with the self did not enhance the salience of their objects over those associated with other people. This result is consistent with previous findings that children (Wuyun et al., 2020) and adults with ASD (Grisdale et al., 2014) do not recall objects associated with the self with superior accuracy, and evidence that children with ASD seemingly disregard their status as owners when valuing objects (Hartley & Fisher, 2018). However, the relatively weak identification accuracy for self-owned objects displayed by our ASD sample contrasts with Hartley et al. (2021), where children with ASD identified their self-owned objects with significantly greater accuracy than objects owned by experimenters and on par with TD children. A likely explanation for this discrepancy concerns differences in the familiarity of self-owned items between these two studies. In Hartley et al. (2021), the objects associated with the self were already owned by the child going into the study (e.g. lunchboxes and items of clothing) and the experimenter’s items were newly introduced to the child, whereas in the present research, both objects associated with the self and objects associated with the experimenter were newly introduced. Hence, children’s increased experience with their self-owned objects in the prior study likely enhanced their identification accuracy.

For children with ASD, associating an object with the self did not induce preferential biases, even when there were no other dimensions on which to base preferences. This result is consistent with previous evidence that children with ASD do not consider randomly endowed objects to be any more valuable than identical copies, which they are often happy to trade for (unlike TD children; Hartley & Fisher, 2018). Interestingly, our data also showed that children with ASD did not drastically differ in their preferences for self-owned items in participant-plain trials (33% preference) in comparison to varied (35%) and identical trials (34%). By contrast, TD children demonstrated a predictable disliking of their assigned objects in participant-plain trials (6%) in comparison to varied (34%) and identical trials (45%). Given that children with ASD reliably inferred that the experimenter preferred their assigned items in participant-plain trials, it seems unlikely that they misunderstood the question or were selecting objects at random. Rather, it could be that objects construed as “undesirable” by TD children were not perceived that way by children with ASD, and their chance-level responding simply reflects the 33.33% likelihood of being assigned their favourite of the three objects on each trial. It is possible that some of the undesirable objects idiosyncratically appealed to children with ASD by providing specific tactile sensorimotor stimulation (Jones et al., 2003), or because their contrast against the two thematically similar toys made them stand out or appear rare (Ferera et al., 2020). In any case, this study provides further evidence that object preferences of children with ASD may qualitatively differ from those of TD children, and additional research is required to elucidate why and how object desirability may differ between these populations.

The owner identification responses and preferences of our children with ASD support theoretical claims that associating objects with the self does not elicit ownership-induced cognitive biases in this population (Hartley & Fisher, 2018; Hartley et al., 2020, 2021; Nijhof & Bird, 2019; Wuyun et al., 2020). In typical development, children’s concept of ownership is thought to arise from extending their psychological sense of self to objects (Diesendruck & Perez, 2015; Rochat, 2010). However, ASD is characterised by differences in the development of self-other understanding, including awareness of mental states (Ben Shalom et al., 2006; Hill et al., 2004; Silani et al., 2008; Williams & Happé, 2010), episodic autobiographical memory (Crane & Goddard, 2008), and the use and comprehension of first-person pronouns (e.g. “I” and “me”; Evans & Demuth, 2012; Overweg et al., 2018; Tager-Flusberg, 1994). Unlike for TD children, information and objects tagged as self-relevant may not occupy a privileged status in the memories of children with ASD (Lind, 2010). Consequently, ownership – which fundamentally constitutes an autobiographical association between an object and the self – may be of reduced psychological importance for children with ASD, and the salience of their property may not be enhanced by the transfer of preferential biases associated with the self.

Apart from children with ASD in participant-plain trials, neither group inferred that the experimenter preferred their assigned items at above chance rates. This broadly aligns with the results of 2- and 3-year-olds, but contrasts with adults (Gelman et al., 2012). It would have been logical for children to infer that the experimenter preferred their objects because they selected the one they liked most at the start of each trial when designating owner-object relationships. It may be that reasoning of this nature would underpin the responding of adult participants, but is contingent on sophisticated mentalising abilities that develop after 4 years (Miller, 2012). Such reasoning abilities may also develop in ASD, as demonstrated by recent evidence that autistic adults understand that other people would be impressed by their ownership of authentic objects (Hartley et al., 2020). Thus, further research is required to pinpoint when and how awareness of others’ ownership-related preferences develops for TD children and children with ASD.

At an applied level, our findings could have implications for social interventions, particularly those that are peer-mediated. Peer-mediated interventions are often designed to promote important social skills in children with ASD, including peer engagement and relationship building (Kasari et al., 2012; Locke et al., 2019). However, reduced understanding and identification of associations between people and property may increase the likelihood of children with ASD interacting with objects without the consent of their owners. Breaching others’ ownership rights in this manner would impact social reputation amongst peers and hinder the development of positive interpersonal relationships. Differences in identifying ownership, and understanding its importance to others, may also contribute to challenging behaviours relating to property destruction that are commonly observed in ASD (e.g. Horner et al., 2002). Furthermore, children with ASD may be at increased risking of exploitation and theft due to their reduced ability to track, and diminished preferences for, self-owned property. We recommend that future studies begin to examine how differences in ownership understanding influence the efficacy of social interventions involving interactions between autistic and TD children.

It is necessary to reflect on the limitations of this research. Firstly, it may be the case that the observed differences between children with ASD and TD children were underpinned by differences in experience (the ASD group were significantly older) or cognitive functioning. Including a group of children with delayed cognitive development matched to our ASD sample on chronological age and nonverbal intelligence would have addressed this issue. Secondly, it is possible that stronger effects of ownership on object preferences may have been observed in both groups had we told children that they could keep the objects they were allocated. Establishing a permanent owner-object relationship may have elicited increased preferences for children’s self-assigned objects as a means of reducing cognitive dissonance (e.g. by aligning their desires with ownership; Elliot & Devine, 1994). Thirdly, it is important to recognise the possibility that more prominent between-population differences may emerge if children were required to independently infer ownership associations between people and property through observing naturalistic behaviour. It would be valuable to investigate how children with ASD interact with owned and non-owned property in social situations with peers and siblings (see O’Brien et al., 2020 and Ross, 2013).

To summarise, this study has provided new insight into how ASD impacts children’s owner identification and object preferences. Our result show that children with ASD may have difficulty tracking invisible historical associations between people and property, irrespective of visual similarity or desirability (although both of these factors may be a hindrance). Our results also show that children with ASD identify novel objects designated to them with no greater accuracy than objects designated to others, and that association with the self does not bias children’s object preferences (even when there are no other dimensions on which preferences could be based). We propose that, due to differences in development of the psychological self, ownership does not increase the attentional or preferential salience of objects for children with ASD. Importantly, differences in ownership understanding may contribute to the social difficulties faced by children with ASD by disrupting their understanding and navigation of relationships between people and property.

## Supplementary Information

Below is the link to the electronic supplementary material.

Supplementary file1 (RTF 56 KB)
