# Supplementary material for: Exploring the Influence of Object Similarity and Desirability on Children’s Ownership Identification and Preferences in Autism and Typical Development
Source: J Autism Dev Disord. 2022 Mar 23;53(6):2362–72. doi: 10.1007/s10803-022-05489-z (PMC10229477; doi:10.1007/s10803-022-05489-z)
Supplement: Supplementary file 1 — Supplementary file1 (RTF 56 KB) [file 10803_2022_5489_MOESM1_ESM.rtf]

Descriptions of variables in file: ASD, Ownership & Object Preferences – data

Population:	Indicates whether the participant has autism spectrum disorder (ASD) or typical development (TD).

Gender:			Indicates whether the participant is “male” or “female”.

Age_Months:			Indicates the participant's chronological age in months.

CARS:	Indicates the participant's raw score on the Childhood Autism Rating Scale (Schopler et al., 2010). Higher scores indicate more severe  autism symptoms.

BPVS_AgeEquiv:	Indicates the participant's age equivalent (in months) on the British Picture Vocabulary Scale (Dunn et al., 1997). 

Varied_Ownership_Child:	Indicates the number of trials (out of 4) that the participant correctly identified their designated object in Varied trials.

Varied_Ownership_Exp:	Indicates the number of trials (out of 4) that the participant correctly identified the experimenter's object in Varied trials.

PPlain_Ownership_Child: 	Indicates the number of trials (out of 4) that the participant correctly identified their designated object in Participant Plain trials.

PPlain_Ownership_Exp: 	Indicates the number of trials (out of 4) that the participant correctly identified the experimenter's object in Participant Plain trials.

Identical_Ownership_Child:	Indicates the number of trials (out of 4) that the participant correctly identified their designated object in Identical trials.

Identical_Ownership_Exp:	Indicates the number of trials (out of 4) that the participant correctly identified the experimenter's object in Identical trials.

Varied_Preference_Child:	Indicates the number of trials (out of 4) that the participant identified their designated object when asked which object they most preferred in Varied trials.

Varied_Preference_Exp:	Indicates the number of trials (out of 4) that the participant identified the experimenter's object when asked which object the experimenter preferred in Varied trials.

PPlain_Preference_Child:	Indicates the number of trials (out of 4) that the participant identified their designated object when asked which object they most preferred in Participant Plain trials.

PPlain _Preference_Exp:	Indicates the number of trials (out of 4) that the participant identified the experimenter's object when asked which object the experimenter preferred in Participant Plain trials.

Identical_Preference_Child:	Indicates the number of trials (out of 4) that the participant identified their designated object when asked which object they most preferred in Identical trials.

Identical _Preference_Exp:	Indicates the number of trials (out of 4) that the participant identified the experimenter's object when asked which object the experimenter preferred in Identical trials.
